# Low rate of function-limiting side effects with high-dose adjuvant radiotherapy in high-grade soft tissue extremity sarcomas: a retrospective single-center analysis over 10 years

**DOI:** 10.1007/s00432-022-04423-1

**Published:** 2022-10-26

**Authors:** Muemtaz Koeksal, Maike van der Hoek, Davide Scafa, David Koch, Christina Leitzen, Leonard C. Schmeel, Georg Feldmann, Andreas Strauss, Sebastian Koob, Frank A. Giordano

**Affiliations:** 1 Department of Radiation Oncology, University Medical Center, Bonn, Germany; 2 Internal Medicine Oncology, Hematology and Rheumatology, University Medical Center, Bonn, Germany; 3 Orthopaedics and Trauma Surgery, University Medical Center, Bonn, Germany; 4 Department of Radiation Oncology, University Medical Center, Mannheim, Germany

**Keywords:** Soft-tissue sarcomas, Extremity sarcomas, Radiotherapy, Adverse events, Local recurrences

## Abstract

**Background:**

Over the years, radiotherapy has been established as a tool to improve local control for high-grade sarcomas. Although the European Society for Medical Oncology guidelines has taken notice of a shift toward a neoadjuvant radiotherapy approach, the American Society for Radiation Oncology guidelines clearly favor a neoadjuvant approach, citing debilitating long-term adverse effects when radiotherapy is applied postoperatively. In this study, we examined these irradiation-associated adverse events for adjuvant radiotherapy and focused on the prognostic factors for disease outcome, including local control.

**Methods:**

In this retrospective study, data for 106 patients with extremity soft-tissue sarcomas diagnosed between 1997 and 2021, of which 40 received adjuvant radiotherapy, were collected from the clinical and radiological information systems of a high-volume sarcoma treatment center. These data were then analyzed for radiation-associated side effects as well as predictive factors for overall survival, disease-free survival, local control, and surgical complications.

**Results:**

Radiotherapy was beneficial to patients improving local control, especially for high-grade sarcomas, even when those were resected with negative margins. Side effects due to radiotherapy occurred in 87.5% of the patients, and these effects primarily included radiation dermatitis in 67.5%; however, only 40.0% had any adverse event of ≥ grade 2 according to Common Terminology Criteria for Adverse Events. Long-term function-limiting side effects occurred in 45.0% of the patients; 10% exhibited ≥ grade 2 function-limiting adverse events. Greater time between surgery and adjuvant radiotherapy was beneficial for the patients, whereas joint infiltrating sarcomas were associated with more severe long term, function-limiting adverse events. 28.3% of the patients experienced a recurrence at any location (median time 18.35 months) and in 16% the recurrence was local (median time 16.11 months), resulting in 1, 3, and 5 year disease-free survival rates of 74.1, 58.9, and 38.5% and local control rates of 78.7, 61.6, and 42.8% were observed, respectively.

**Conclusion:**

Recurrences may be avoided with high-dose radiation, especially for high-grade G2 and G3 sarcomas, even after complete R0 resection. This resulted in a low rate of severe long-term function-limiting adverse events. Thus, adjuvant radiotherapy should be seriously considered when planning patient treatment, especially when treating patients that present with high-grade sarcomas.

**Supplementary Information:**

The online version contains supplementary material available at 10.1007/s00432-022-04423-1.

## Background

Soft-tissue sarcomas are a very rare type of mesenchymal tumors that account for < 1% of adult solid malignancies (Burningham et al. 2012). In the United States in 2022, 13,190 people are expected to be diagnosed with soft-tissue sarcomas and 5130 are expected to die from this disease (Key statistics for soft tissue sarcomas 2022). This tumor type encompasses different histological entities, for example, leiomyosarcomas and liposarcomas, which can form all over the body, but most often occur in the extremities. The heterogeneity combined with the rarity of sarcomas is considered a challenge for research and treatment.

Over the last decades and since Rosenberg et al. demonstrated that complete limb amputation is not necessary for successful treatment (Rosenberg et al. 1982), a multimodal approach has been implemented. Currently, therapy poses an interdisciplinary challenge involving surgery, chemotherapy (CHT), and radiotherapy (RT), which should be administered at high-volume centers (with ≥ 10 soft-tissue sarcomas per year) (Abarca et al. 2018). This has resulted in survival rates increasing and the 5 year overall survival (OS) now standing at approximately 70% (Al-Absi et al. 2010). Notably, the rate of local recurrences is ~ 15% for soft-tissue extremity sarcomas (STES), and most of these occur within 2 years (Eilber et al. 2005).

Over the years, it has been demonstrated that negative surgical margins remain a key metric to prevent local recurrence and improve OS (Trovik et al. 2000; Vraa et al. 2001; Dickinson et al. 2006; Novais et al. 2010; Gronchi et al. 2007). Owing to the higher associated risk (Jebsen et al. 2008), patients with G2- and G3-rated sarcomas or with positive margins require RT to improve local control (LC) (Leitlinie and Weichgewebesarkome. 2022). Nonetheless, RT, which remains a beneficial tool for treating patients to improve LC and OS, remains underused (Bagaria et al. 2014).

Moreover, there is no definite consensus on whether to use neoadjuvant (neoadj.) or adjuvant (adj.) RT because both modalities have their own advantages and adverse effects. Nevertheless, both options improve OS (Ramey et al. 2018). In the US, RT before surgery remains the standard, whereas the interval between RT and surgery is still debatable. In Europe, patients mostly undergo adjuvant RT (Hoefkens et al. 2016) (2016), although the European guidelines established by ESMO describe the increasing use of preoperative RT (Gronchi et al. 2021), whereas the American clinical guidelines by ASTRO clearly favor the preoperative approach to reduce the side effects associated with the high-dose adj. RT (Salerno et al. 2021). These issues were the subject of other studies pioneered by the CAN-NCIC-SR2 study, which showed a reduction in late toxicities when RT was administered preoperatively (O’Sullivan et al. 2002), requiring a lower dose than adj. RT. Generally, complications, RT- and surgery-related, can lead to a lower quality of life for patients and should, therefore, be avoided.

One approach consists of the use of intensity-modulated radiation therapy (IMRT), which may be applied very precisely and can spare healthy tissues more effectively compared with conventional RT. Therefore, studies reported that IMRT resulted in a reduction of late toxicities (Demetri et al. 2005). Using closer security margins for neoadj. RT, Wang et al. reported that the reduction in irradiation margins was safe and resulted in a reduction in late toxicities (Wang et al. 2015).

Regarding the use of RT in European high-volume sarcoma treatment facilities, patients are mostly treated with postoperative RT within an interdisciplinary approach. In this study, we examined the aforementioned adverse side effects that occur with adj. RT and assessed the risk of wound complications and disease outcomes, including OS and local recurrences, in this patient cohort.

## Methods

### Overview

Data were collected from clinical and radiology information system of our university medical sarcoma center as well as the German Centre for Cancer Registry. Patients were identified using the search word “sarcoma” from the data of those who had received any treatment within the last 10 years and were diagnosed with an extremity soft-tissue sarcoma, except for cutaneous sarcomas. The data of patients who had undergone adj. RT and for which the follow-up deadline was February 2nd, 2022 were assessed. Subsequently, a retrospective analysis of the data was performed.

For all patients, the diagnosis of sarcoma was confirmed using histological analysis after biopsy or primary surgery, and the patient’s age at that date was recorded. After diagnosis, treatment decisions were made by an interdisciplinary team with patients’ informed consent.

Resection margins were classified according to quality and categorized into three groups: R0 (microscopically negative margins), R1 (microscopically positive margins) and R2 (macroscopically positive margins). For RT, irradiation doses were prescribed to cover 99% of the clinical target volume (CTV), 95% of the PTV, and ranged from 95%–107% of the prescribed dose. The biologically effective irradiation dose (BED) and the equivalent total dose in 2 Gy fractions (EQD2) were calculated with the α/β ratio, which was considered to be 4 (Leeuwen et al. 2018).

**Fig. 1:**
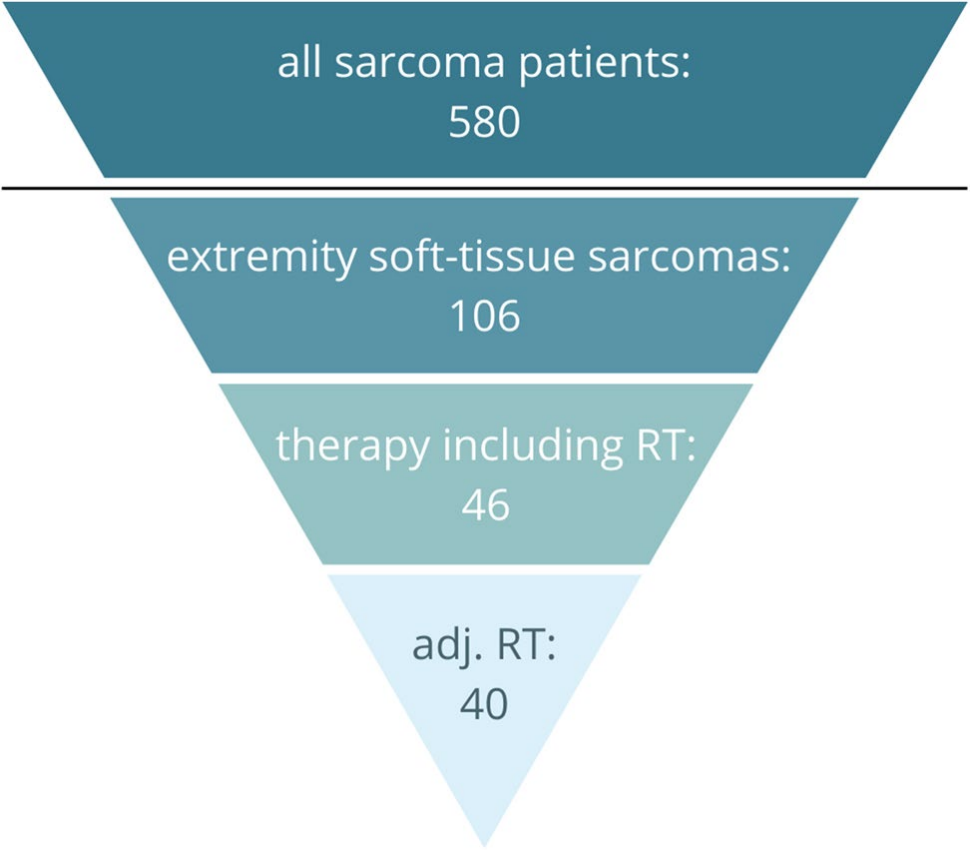
Selection of patients

**Fig. 2:**
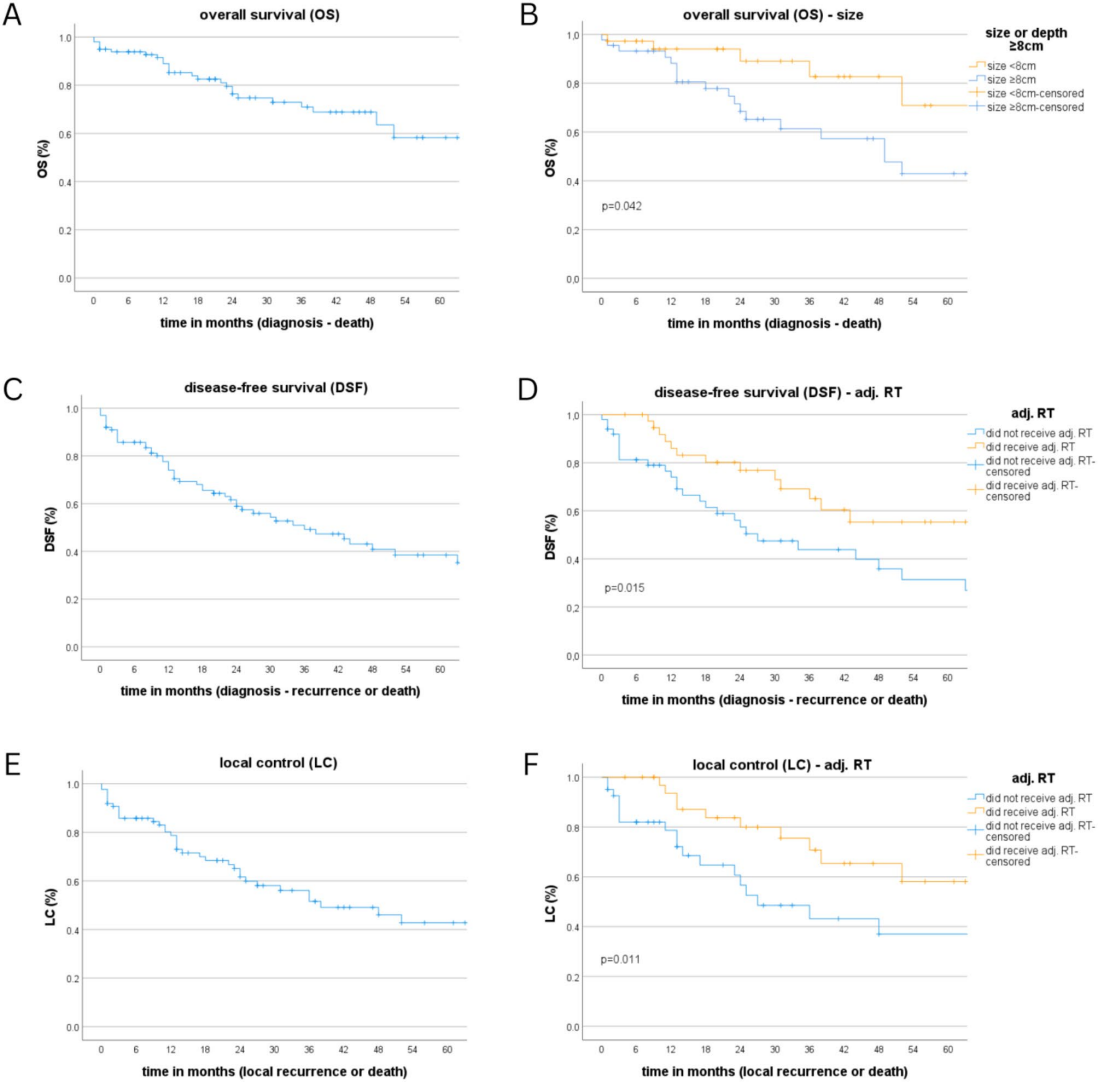
Kaplan–Meier graphs for overall survival, disease-free survival, and local control. **A** OS, **B** OS compared in terms of the size. **C** DSF, **D** DSF compared in terms of the adj. RT. **E** LC, **F** LC compared in terms of the adj. RT

**Fig. 3:**
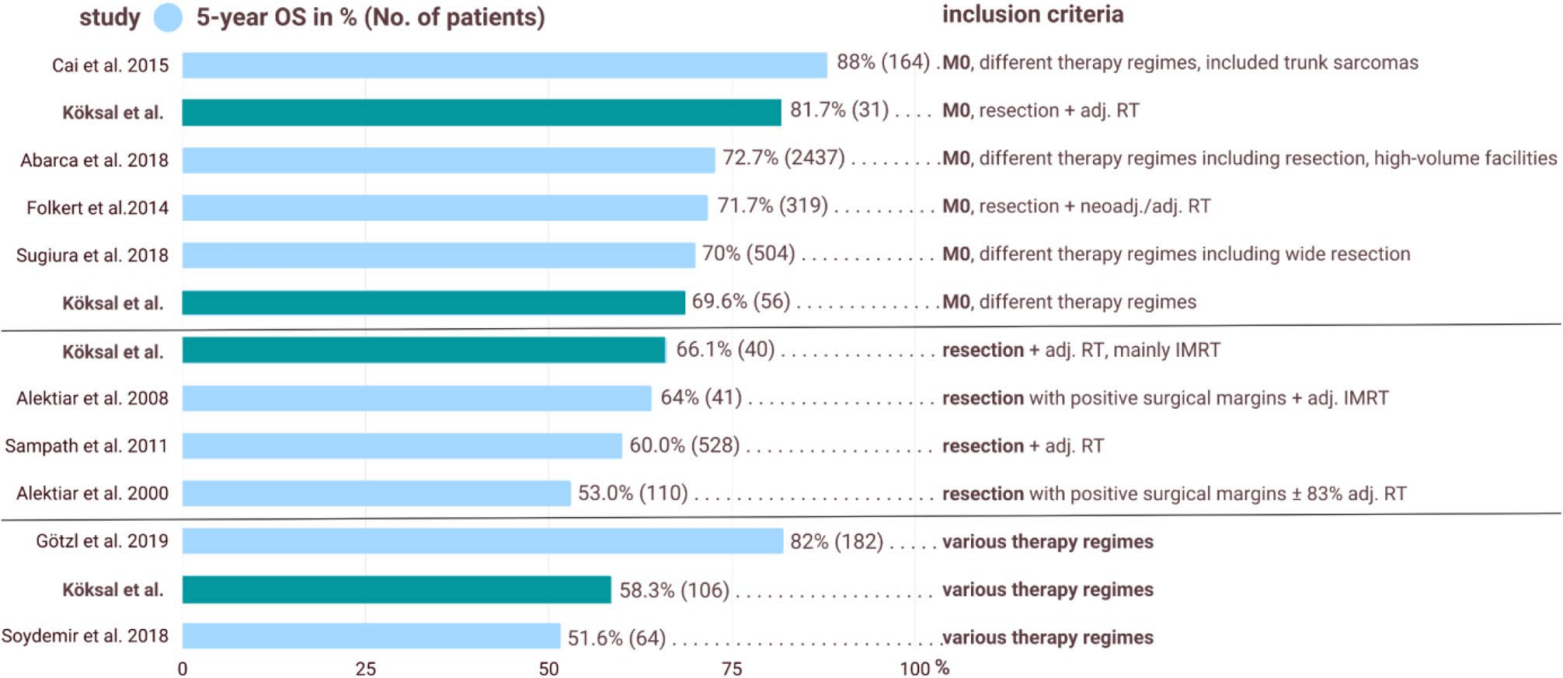
Comparison of 5 year OS: different studies presented with their observed 5 year OS and the number of patients included, as well as inclusion criteria. *OS* overall survival, *M0* absence of metastatic disease, neoadj./adj. *RT* neoadjuvant/adjuvant radiotherapy, *IMRT* intensity-modulated radiation therapy

Patients were followed at radiotherapy-specific check-ups as well as in the clinic. Follow-up reports also provided data on RT adverse events classified by the Common Terminology Criteria for Adverse Events (CTCAE) (Common terminology criteria for adverse events (CTCAE) 2017) and surgery complications. Recurrences detected in the clinical examination were confirmed by CT or MRI and histology. Patients, sarcoma, and treatment characteristics are summarized in Table 1, Fig. 1, and the supplementary material (SM) Figs. 1, 2, 3.

**Table 1:** Patient and tumor characteristics. G1 = low-grade, G2 = intermediate-grade, G3 = high-grade

Characteristic	All (106)	% (*n* = 106)	Adj. RT	% (*n* = 40)
Age, years				
Median	55.18		56.50	
Range	0–91		22–85	
< 70 years	77	72.6%	29	72.5%
≥ 70 years	29	27.4%	11	27.5%
Sex				
Male	57	53.8%	17	42.5%
Female	49	46.2%	23	57.5%
Location of sarcoma				
Upper extremity	21	19.8%	5	12.5%
Upper arm	11	10.4%	4	10.0%
Forearm/hand	10	9.4%	1	2.5%
Lower extremity	85	80.2%	35	87.5%
Thigh	51	48.1%	23	57.5%
Lower leg/foot	14	13.2%	6	15.0%
Hip or buttocks	20	18.9%	6	15.0%
Histology				
Undifferentiated (pleomorphic) sarcoma	40	37.7%	12	30.0%
Synovial sarcoma	12	11.3%	5	12.5%
Liposarcoma	8	7.5%	6	15.0%
Myxofibrosarcoma	8	7.5%	7	17.5%
Leiomyosarcoma	7	6.6%	2	5.0%
Rhabdomyosarcoma	5	4.7%	1	2.5%
(Extraskeletal myxoid) chondrosarcoma	5	4.7%	1	2.5%
MPNST	4	3.8%	2	5.0%
Others	15	14.2%	2	5.0%
Unclassified	2	1.9%	2	5.0%
Histological grade				
G1	9	8.5%	3	7.5%
G2	17	16%	10	25.0%
G3	57	53.8%	25	62.5%
Unknown	23	21.7%	2	5.0%
Size (longest axis)				
≤ 5 cm	20	18.9%	6	15.0%
> 5 cm	68	64.2%	31	77.5%
Exact size unknown	18	17%	3	7.5%
Recurrences				
All recurrences	30	28.3%		
Distant recurrences	13	12.3%		
Local recurrences	17	16.0%		
Local recurrences after receiving adj. RT			4	10.0%
Median time from diagnosis to recurrence (any)	18.35	Months		
Median time from diagnosis to local recurrence	16.11	Months		
Deaths	31	29.2%	9	22.5%
Death due to localized disease	0	0%	0	0.0%
Death due to metastatic disease	13	12.3%	4	10.0%
Death from treatment complications	1	0.9%	1	2.5%
Death due to any other reasons or reason unknown	17	16%	4	10.0%

### Statistical endpoints and methods

Descriptive statistics were used to analyze patient and tumor characteristics as well as therapy regimens, toxicity, and recurrences. The main endpoint of this study was adverse effects following adj. RT as defined by CTCAE criteria [Common terminology criteria for adverse events (CTCAE) 2017]. The impact of various factors was determined using binary logistic regression models. The secondary endpoints included OS, which was defined as the time from histological diagnosis to the date of last follow-up or death regardless of the cause. Disease-free survival (DFS) was calculated from the date of diagnosis to recurrence or death. Events for local control (LC) included local recurrences or death from any cause. These time-to-event endpoints were estimated using the Kaplan–Meier method [Kaplan and Meier 1958] and subgroups were compared using the Log-rank test. Patients still alive at the end of follow-up were considered censored. Cox proportional hazards regression was done to assess prognostic factors associated for OS, DFS, and LC. Acute wound complications (defined as requiring secondary operations/invasive procedures for wound care, use of vacuum-assisted closure, prolonged dressing changes, or infection within 120 days of surgery) were assessed using binary logistic regression. A *p* value of < 0.05 was considered statistically significant. All statistical analyses were performed using IBM^®^ SPSS^®^ software (version 28.0.1.1; IBM Corp., Armonk, NY, USA).

## Results

Overall, 106 patients with STES were identified and treated with different therapeutic regimens at this institution and their diagnosis was established between 1997 and 2021. The mean follow-up period was 41.28 months (median: 26 [range, 1–288] months). The median age at diagnosis was 55 years, and sarcomas occurred most frequently on the lower extremity (80.2%), especially on the thigh (48.1%). Regarding analysis in terms of histological groups, the most common tumors were undifferentiated (pleomorphic) sarcomas (37.7%), followed by synovial sarcoma (11.3%) and liposarcoma (7.5%).

Histologically, most sarcomas were classified as G3 (57, 21.7%). G1- and G2-rated sarcomas accounted for 8.5 and 16%, respectively. For 21.7% of patients, the grading was unknown and could not be found in the clinical history. Regarding tumor size, 64.2% of tumors were > 5 cm in size, 18.9% were ≤ 5 cm, and 17% were of unknown size.

Surgery was performed on 92 patients (86.8%). Four patients received neoadj. RT, and 40 patients underwent surgery followed by adj. RT. The median time between surgery and adj. RT was 57 days with a mean total irradiation dose of 59.47 Gy, from which a mean BED of 88.14 Gy and an EQD2 of 59.08 Gy was calculated. Therapy-related details are provided in Table 2.

**Table 2:** Therapeutic regimen details

Therapy regime details	No	%	*n*
Surgery	92	86.8	106
amputation	7	7.6	92
limb-sparing surgery	85	92.4	92
direct closure	64	75.3	85
flap closure	7	8.2	85
R0	59	64.1	92
R1	22	23.9	92
R2	5	5.4	92
R unknown	6	6.5	92
no surgery	14	13.2	106
Chemotherapy	42	39.6	106
neoadj. chemotherapy	16	15.1	106
adj. chemotherapy	26	24.5	106
def. chemotherapy	8	7.5	106
no chemotherapy	64	60.4	106
Radiotherapy	46	43.4	106
no radiotherapy	60	56.6	106
neoadj. radiotherapy	4	3.8	106
def. radiotherapy	3	2.8	106
adj. radiotherapy	40	37.7	106
Median time between surgery and adj. RT	57 days		
Mean dose	59.47 Gy		
IG-/IMRT	24	60	40
IGRT	2	5.0	40
Others, including VMAT	3	7.5	40
Unknown	11	27.5	40

*R0* negative margins, *R1* microscopically positive margins, *R2* macroscopically positive margins, *IG-/IMRT* Image-guided/intensity-modulated radiation therapy, *IGRT* Image-guided radiation therapy using Cone-Beam CT, *IMRT* intensity-modulated radiation therapy, *VMAT* Volumetric intensity-modulated arc therapy

In total, 31 patients (29.2%) had died by the follow-up deadline and 13 (12.3%) died from metastatic disease. For 17 patients (16%), the cause of death was unknown. There was only one patient who died from a treatment complication which was CHT-related.

### OS

For the 106 patients, the 1, 2, and 5 year OS were estimated at 89, 76.4, and 58.3%, respectively. The visual representation of all time-sensitive endpoints in the form of Kaplan–Meier graphs is presented in Fig. 2. Negative prognostic patient and tumor factors included lymph node involvement (*p* < 0.001), vascular invasion (*p* = 0.003), metastases (*p* < 0.001) and a tumor size ≥ 8 cm (*p* = 0.042). Surgery was determined to be an important component of therapy (*p* < 0.001). Adjuvant radiotherapy, regardless of tumor stage, and size failed to exhibit a significant impact on OS (*p* = 0.397); however, if adj. RT was received, the total irradiation dose, which translated to a higher BED (*p* = 0.028, HR = 0.969, 95% CI: 0.942–0.997) and a higher EQD2 (*p* = 0.022, HR = 0.953, 95% CI: 0.915–0.993) had a positive impact (*p* = 0.016, HR = 0.952, 95% CI: 0.915–0.991). No prognostic value was established for the location of the sarcoma (location in general, upper vs. lower and proximal vs. distal extremity) or joint involvement at the time of diagnosis.

### RT adverse events

Forty-six patients received RT during treatment; of these, 40 received RT postoperatively. Recorded adverse events included joint stiffness after radiation, edema, fatigue, pain, hyperpigmentation, wound healing disorders, hardened soft tissue, and RT-associated colitis. Thirty-five patients (87.5%) experienced at least one of adverse effect with 16 (40.0%) having had one, which was scored at ≥ grade 2 according to CTCAE. The most common adverse event was radiation dermatitis, which affected 27 patients (67.5%). With respect to the long-term function-limiting adverse events, joint stiffness, edema, and tissue fibrosis of any grade occurred in 45.0% of the patients. For ≥ grade 2, the rate was 10% (Table 3).

**Table 3:** RT adverse events sorted by severity according to CTCAE

RT adverse events	No	% of *n* = 40
patients with radiation side effects	35	87.5
patients with ≥ 2nd grade	16	40.0
patients with ≥ 3rd grade	5	12.5
patients with ≥ 4th grade	0	0.0

Detailed listings of RT adverse effects	No. CTC° 1/2/3/4	% of *n* = 40
Long term: edema	12/2/1/0	37.50
Long term: joint stiffness	2/0/1/0	7.50
Long term: hardened soft tissue	2/1/0/0	7.50
Radiation dermatitis	15/10/2/0	67.50
Pain	8/1/1/0	25.00
Hyperpigmentation	6/0/0/0	15.00
Fatigue	2/1/0/0	7.50
RT colitis	1/0/0/0	2.50
Wound healing disorder after adj. RT	3 in total	7.50

Analyzing possible predictors of adverse effects in general, a longer time between surgery and adj. RT was beneficial with adverse effects being less likely each day (*p* = 0.013, HR = 0.984, 95% CI: 0.972–0.997). The timing, however, did not have an effect on the amount of wound healing disorders after receiving adj. RT (7.5%) (*p* = 0.784). When only considering the aforementioned long-term function-limiting events, sarcomas that infiltrated the adjacent joint were 11 times more likely to result in more severe function-limiting ≥ grade 2 events (*p* = 0.040, 95% CI: 1.115–108.448).

The other possible predictors (sex, age, age under 70, sarcoma location, upper vs. lower extremity sarcoma, histology, lymph node involvement, metastasis, tumor grade, stage, lymph node and blood vessel invasion, multifocality, tumor size, having undergone neoadj. therapy options, such as CHT and RT, and resection status) failed to have a significant impact. Moreover, there was no association for radiotherapy parameters, including technique, total dose, dose per fraction, BED, and EQD2.

### DFS and LC

Of 106 patients reported in this study, 30 (28.3%) experienced a recurrence. The median time from the date of diagnosis to the discovery of recurrence, regardless of location, was 18.35 months. Regarding local recurrences, the time was reduced to 16.11 months. The recurrence rates for the lower and upper extremity sarcomas were 25.88 and 33.33%, respectively. Among the 17 patients with local recurrences, 6 had previously positive resection margins (R1 or R2) after surgery. Ten local recurrences occurred in patients with negative margins (R0). Only four patients who had received adj. RT for LC experienced local recurrences. All these recurrences occurred in the lower extremity, and 3 of 4 occurred within the 90% isodose. The same three patients had previously undergone R1 resections. The last recurrence occurred within the 25% isodose after a previous R0 resection (SM Table 1, SM Fig. 4).

For all recurrences, the estimated DSF rates for 1, 2, 3, and 5 years were 74.1, 58.9, 49.2, and 38.5%, respectively, and 1, 2, 3, and 5 year LC rates were 78.7, 61.6, 51.6, and 42.8%, respectively. Patient’s age of ≥ 70 years had a negative effect (DSF: *p* = 0.19, LC: *p* = 0.010); in addition, lymph node involvement (DSF: *p* = 0.004, LC: *p* = 0.001), vascular invasion (DSF: *p* = 0.23, LC: *p* < 0.001), metastases (*p* < 0.001), no adj. RT after surgery (DSF: *p* = 0.19, LC: *p* = 0.11), and no surgery (*p* < 0.001) demonstrated negative effects. No significant prognostic value was found for location, joint involvement, histological group, stage, or size.

The inclusion of adj. RT in treatment regimen for high-grade sarcomas was found to be significantly beneficial compared with no adj. RT treatment regimen (G2 and G3) (DSF: *p* = 0.012, LC: *p* = 0.026); however, this was not evident while considering only G1-rated sarcomas (G1). Notably, only eight patients had G1-sarcomas. For LC, adj. RT was beneficial even for patients who underwent a complete R0 resection (*p* = 0.018); however, this primarily included those with G3 sarcomas and only a few with G1-sarcomas (G1: 5, G2: 8, G3: 31, unknown: 6). Regarding G2 and G3 sarcomas after complete R0 resection, adj. RT was still significantly beneficial (*p* = 0.030).

A more detailed analysis of adjuvant radiotherapy revealed that a higher total irradiation dose, BED, and EQD2 were associated with fewer recurrences in general (*p* = 0.26, HR = 0.963, 95% CI: 0.931–0.995; *p* = 0.43, HR = 0.976, 95% CI: 0.953–0.999; *p* = 0.029, HR = 0.963, 95% CI: 0.930–0.996). EQD2 remained significant when adjusted for tumor grade and resection margin (*p* = 0.020).

The same adj. RT parameters were found to have an impact on LC, including a higher total dose (*p* = 0.024, HR = 0.961, 95% CI: 0.928–0.995), BED (*p* = 0.042, HR = 0.975, 95% CI: 0.951–0.999), and EQD2 (*p* = 0.029, HR = 0.960, 95% CI: 0.926–0.996), even when adjusted for grade and resection margin (*p* = 0.012, HR = 0.949, 95% CI: 0.910–0.988). No significant association was observed for time between surgery and adj. RT (*p* = 0.199).

### Surgery-related complications

Of 92 patients having undergone surgery, 23 (25.0%) required a secondary operation or other invasive procedure for wound care, whereas 7 patients (7.6%) required prolonged dressing changes and 7 (7.6%) experienced wound infections within 120 days from the date of surgery. For five patients (5.4%), vacuum-assisted closure (VAC) was used. When wound complications and VAC were combined, 29.3% were experienced at least one of these complications (SM Table 2).

Of 70 patients with lower extremity sarcomas having undergone surgery, 22 (31.4%) experienced complications, whereas 5 (23.8%) of 21 patients received surgery for upper extremity sarcomas.

Prognostic factor analysis for complications revealed that the size of the preoperative tumor was significant. A tumor size or depth of ≥ 8 cm was 2.88 times more likely to cause complications (*p* = 0.047, 95% CI: 1.015–8.180), whereas the Hazard ratio for a size of ≥ 10 cm was 2.93 (*p* = 0.038, 95% CI: 1.062–8.056). No significance was found with respect to location in general, upper vs. lower extremity, proximal vs. distal extremity, as well as having received neoadj. CHT (any regimen or Doxorubicin/Ifosfamide compared with others).

One in four patients who received neoadj. RT had surgical complications, whereas the other three had none. The mean time between neoadj. RT and surgery was 62.25 days. Owing to the small sample size, analysis of this did not yield significant results.

Regarding the group that received adj. RT, 35% underwent a secondary procedure, 7.5% required prolonged dressing changes, 15% had infections, and 7.5% had their wounds closed with VAC before adj. RT. Importantly, 7.5% of patients experienced wound complications that occurred after adj. RT.

## Discussion

We included 106 patients with STES receiving different treatment regimens with a mean age of 55 years and a mean follow-up of 26 months. Of these, 37.7% received adj. RT, mostly IMRT.

For all patients, the 1, 2, and 5 year OS was 89, 76.4, and 58.3%, respectively; this was consistent with literature, and it is compared in Fig. 3 based on different inclusion criteria. Regarding adj. RT, total exposure dose had a positive impact on OS and resulted in higher BED and EQD2. This should be considered in the light of the side effects and should always be based on the quality of life of the patient.

At least one adverse event of any grade occurred in 87.5% of the patients with the most common being radiation dermatitis for 67.5% of patients that underwent adj. RT. In total, 40.0% of the patients experienced a severe adverse event ≥ grade 2 according to CTCAE.

Demitri et al. (2005) reported a significant decrease in late toxicities, especially edema and joint stiffness, with the use of the precise method of IMRT instead of conventional RT. IMRT use has significantly increased over the last decades and was the primary RT technique used in this study (60%). Thus, similar to this study, IMRT is currently considered the primary RT technique.

It has been suggested that adj. RT results in more long-term function-limiting side effects compared with neoadj. RT, especially joint stiffness, edema and fibrosis (Salerno et al. 2021; Cammelli et al. 2021). In the present study, 45.0% of patients experienced at least one of the three of any grade sarcoma significantly impacting their quality of life (O’Sullivan et al. 2002). However, this was only 10% (7.5% for severe edema and 2.5% each for severe joint stiffness and tissue fibrosis) for ≥ grade 2, which compares favorably with the results of other studies (Table 4). A possible underreporting error because of the retrospective nature of this study should be considered. Regarding the long-term function-limiting events, joint infiltrating sarcomas had a risk of more severe events (≥ grade 2). Nonetheless, it is clear that adj. RT remains an important option for the treatment of sarcoma patients with severe long-term side effects being ameliorated.

**Table 4:** Comparison of adj. RT adverse-events-rate to other studies

Study	No of patients, RT	Edema ≥ 2nd grade	Joint stiffness ≥ 2nd grade	Fibrosis ≥ 2nd grade
Beane et al. (2014)	28 adj. RT	25%	10%	Not reported
Alektiar et al. (2008)	7 neoadj., + 34 adj. RT	12.2%	17.1%	Not reported
Köksal et al	40 adj. RT	7.5%	2.5%	2.5%
Davis et al. (2005)	56 adj. RT	25.0%	23.2%	48.2%
Folkert et al. (2014)	319 adj. RT	11.3%	12.9%	Not reported

In general, a longer time between surgery and adj. RT was beneficial; however, the timing did not have an effect on the amount of wound healing disorders after receiving adj. RT or local recurrences. No significant impact was evident for adj. RT parameters; therefore, no conclusion can be drawn regarding the effect of hypofractionated RT on the side effects of RT; however, the results by Lee et al. with no correlation for RT dose and field size (Lee et al. 2012) can be confirmed.

In this study, the rate of recurrences, one of the negative prognostic factors for OS (Alektiar et al. 2011), was 28.3%. Local recurrences occurred in 16.0%, and these mostly occurred in patients with previous negative margins. Therefore, the 1, 2, and 5 year rates were 74.1, 58.9, and 38.5% for DFS, and 78.7, 61.6, and 42.8% for LC, respectively. Similar to Alektiar et al.’s study (Alektiar et al. 2008), sarcoma size and grade had no impact on LC. Conversely (Alektiar et al. 2000), no prognostic value was observed in terms of location. Negative prognostic factors included age of ≥ 70 years, lymph node involvement, vascular invasion, metastatic disease at diagnosis, absence of surgery, and absence of RT. Regarding OS, surgery with negative margins is extremely important when considering positive margins as one of the main prognostic factors of recurrences (Vraa et al. 2001; Dickinson et al. 2006; Novais et al. 2010; Gronchi et al. 2007).

Not having undergone RT as a prognostic factor also reported elsewhere in literature (Yang et al. 1998) demonstrates the importance of this therapy and suggests that RT should be seriously considered when planning patient treatment. This effect is especially important for high-grade sarcomas as Alektiar et al. (2000) reported the beneficial effects after previous positive surgical margins in high-grade sarcomas. However, Jebsen et al. observed a significant effect on low-grade sarcomas and after wide resections (Jebsen et al. 2008). We could not reproduce the effect for low-grade sarcomas possibly because of the small sample size. Nonetheless, the benefit of adj. RT after R0 resection remains, but this has to be considered with caution because of the high rate of G3-rated sarcomas. For high-grade sarcomas with R0 resection, a significant benefit was retained. We could also report that higher adj. irradiation doses are associated with fewer recurrences. Delaney et al. revealed that patients with positive margins receiving more than 64 Gy dose had a better 5 year OS, DFS, and LC (85, 52.1, and 67.8%, respectively) (Delaney et al. 2007), as seen in our study. This indicates that higher irradiation doses should be aimed for, of course, considering the possible discussed side effects.

As only four patients received neoadj. RT, we could not conclude on the effects of neoadj. RT like Sampath et al. (Sampath et al. 2011) and Al-Absi et al. (Al-Absi et al. 2010). Wang et al. reported 5 local recurrences out of 74 patients who received neoadj. IGRT, all within the 95% isodose. This demonstrates the safety of reduced longitudinal CTV margins in neoadj. RT (Wang et al. 2015). In the present study, with adj. RT, three of four recurrences appeared within the 90% isodose.

We observed a median time of 16.11 months for local recurrences compared with 18 months reported by Folkert et al. (2014), which may be due to the specific patient cohort. They also showed that IMRT had a significant benefit in the prevention of recurrences compared with conventional RT, which confirms the results of Alektiar et al. (2011). The irradiation dose for IMRT is applied with extreme precision and conformality (Griffin et al. 2007; Stewart et al. 2009); thus, it is beneficial to LC without the undesirable side effects (see above).

Generally, the rate of wound complications and VAC observed in our cohort was 29.3%, with 25% requiring secondary interventions. When considering the higher rate of wound complications with neoadj. RT (O’Sullivan et al. 2002; Wang et al. 2015; Beane et al. 2014; Peeken et al. 2019; Peat et al. 1994), it has been suggested that neoadj. RT results in more wound complications; therefore, postoperative RT should be considered. For example, Götzl et al. reported a complication rate of 28% for neoadj. RT, 8% for adj. RT, and a resulting lower quality of life when compared with neoadj. RT against no RT (Götzl et al. 2019). The 8% is comparable to 7.5% for wound complications after adj. RT in the present study, although different complications were observed. Because of the low number of patients, no conclusion can be made for neoadj. RT.

The main prognostic factor for developing wound complications was tumor size, with a size ≥ 8 cm and ≥ 10 cm being significantly more likely to result in complications, which is consistent with the results of O’Sullivan et al. (2002) and Peat et al. (1994). In contrast (O’Sullivan et al. 2002; Rene et al. 2021), no impact was found regarding sarcoma location with a rate of 31.4% for leg and 23.8% for arm. Further single-center studies are needed to determine if this is due to clinical parameters or patient samples. Positively consistent with other studies, the occurrence of wound complications did not affect LC, DFS, or OS (Rene et al. 2021; Rosenberg et al. 2013).

### Limitations

The limitations of this study include the small patient size as well as the heterogeneity of the sarcomas. In addition, the retrospective design of the study may imply that adverse events related to therapy and patients who are followed up in different hospitals are under-reported and therefore lost in this study. Some follow-ups were conducted by telephone because of the Covid-19 pandemic; this could have resulted in adverse events not being reported with sufficient details. Despite its limitations, this study provides insight into the importance and tolerance of adj. RT within an interdisciplinary approach that includes surgery and RT.

## Conclusions

In this study, we aimed to evaluate adj. RT, which consisted of IMRT in most patients. This was highly beneficial for disease control, especially for high-grade sarcomas, even after complete R0-resections. Higher adj. irradiation doses resulted in better OS and fewer recurrences, and this should be considered while planning patients’ therapy. However, these doses or the dose per applied fraction did not statistically affect the amount of irradiation-induced adverse events. The rate of long-term function-limiting adverse events was lower than that of other studies, indicating that adj. RT should be considered in such patients. As sarcomas infiltrating the joint have serious side effects, special attention should be paid to prevention to this group.

With a 5 year OS of 58.3%, DFS of 38.5%, and LC of 42.8% and a median time to recurrence of 18 months, the need for regular follow-up examinations was demonstrated.

## Supplementary Information

Below is the link to the electronic supplementary material.

Supplementary file1 (DOCX 522 KB)

## Data Availability

The data that support the findings of this study are available from the corresponding author upon reasonable request.
