# Supplementary material for: Low rate of function-limiting side effects with high-dose adjuvant radiotherapy in high-grade soft tissue extremity sarcomas: a retrospective single-center analysis over 10 years
Source: J Cancer Res Clin Oncol. 2022 Oct 26;149(8):4877–88. doi: 10.1007/s00432-022-04423-1 (PMC9607852; doi:10.1007/s00432-022-04423-1)
Supplement: Supplementary file 1 — Supplementary file1 (DOCX 522 KB) [file 432_2022_4423_MOESM1_ESM.docx]

Supplementary materials

**Figures**

###### SM Figure 1: Patients’ age distribution

######
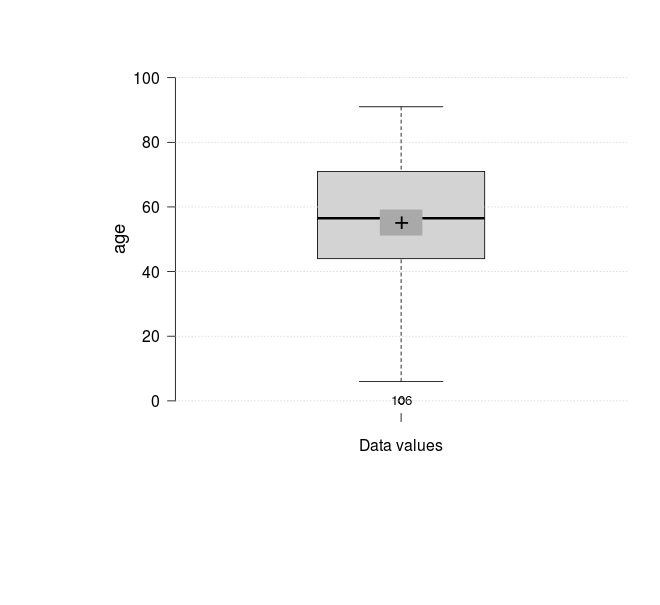


###### SM Figure 2: Location distribution of sarcomas included
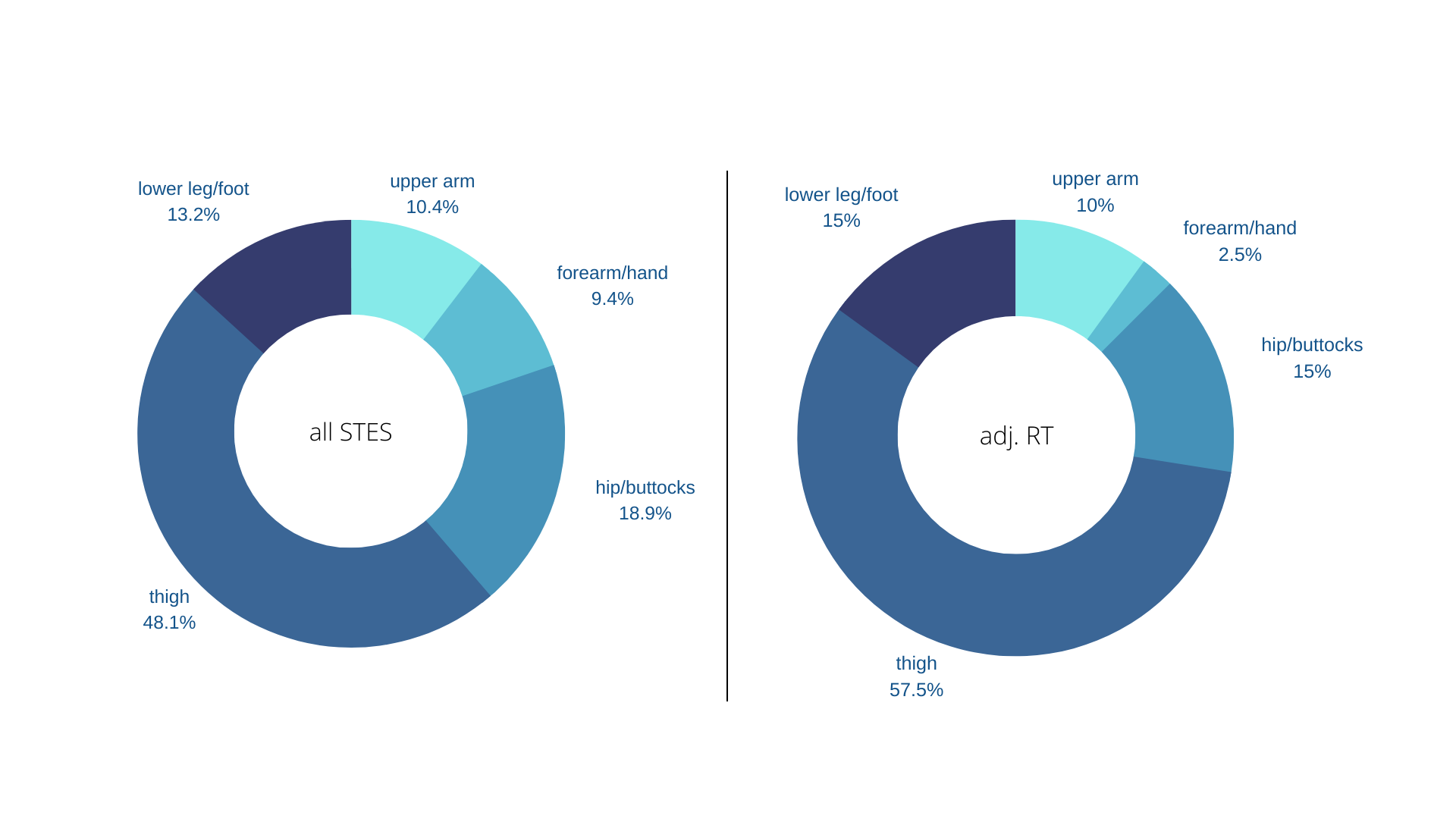


###### SM Figure 3: Histological distribution of sarcomas included

#####
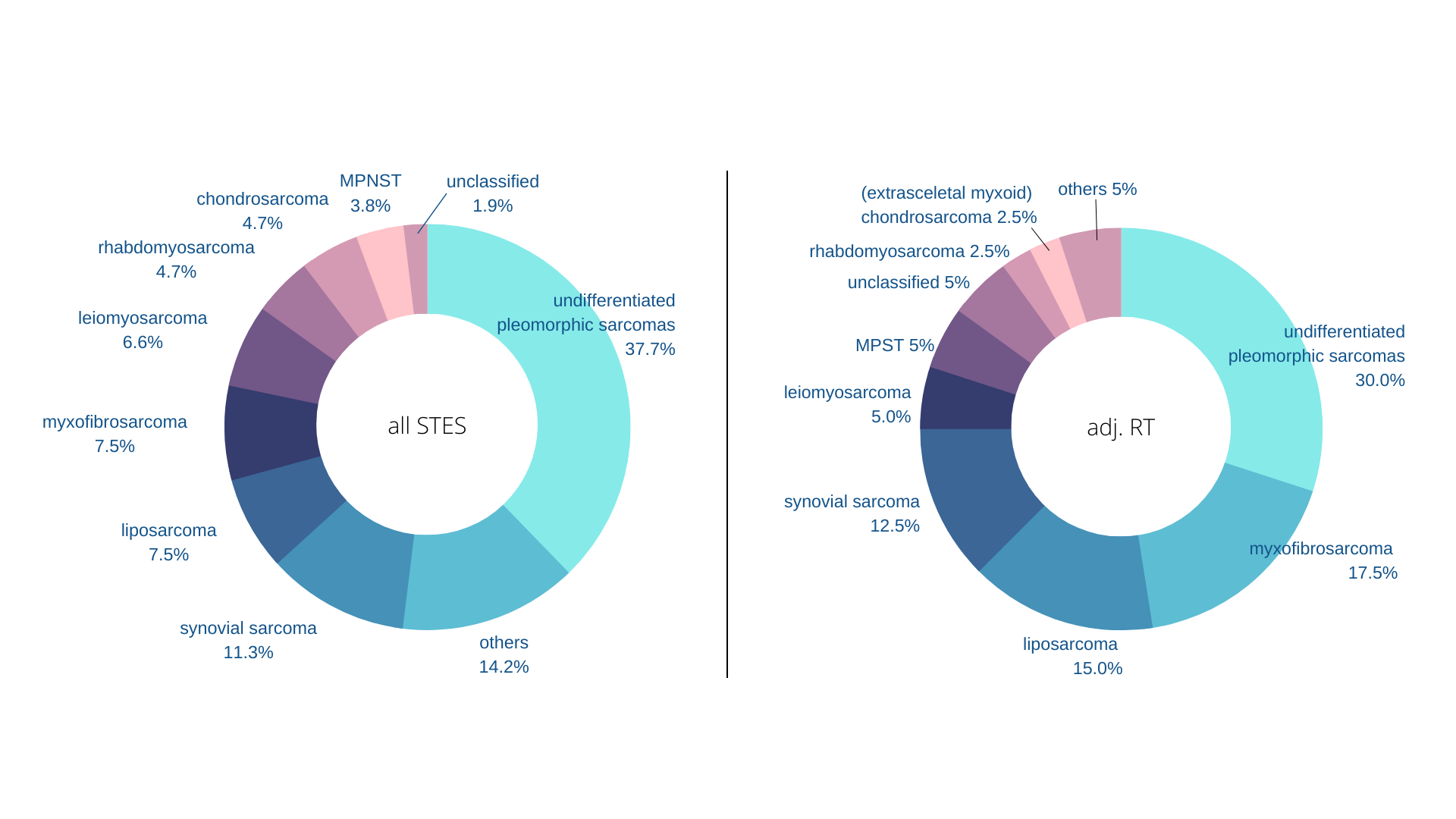


##### *SM Figure 4: MRI of the 56-year-old patient with a synovial sarcoma having received IG-/IMRT RT as an example of the recurrence in relation to the previous irradiation field and its isodoses. This recurrence reaches the 25% isodose (of 60 Gy, see SM Table 1).*

#####
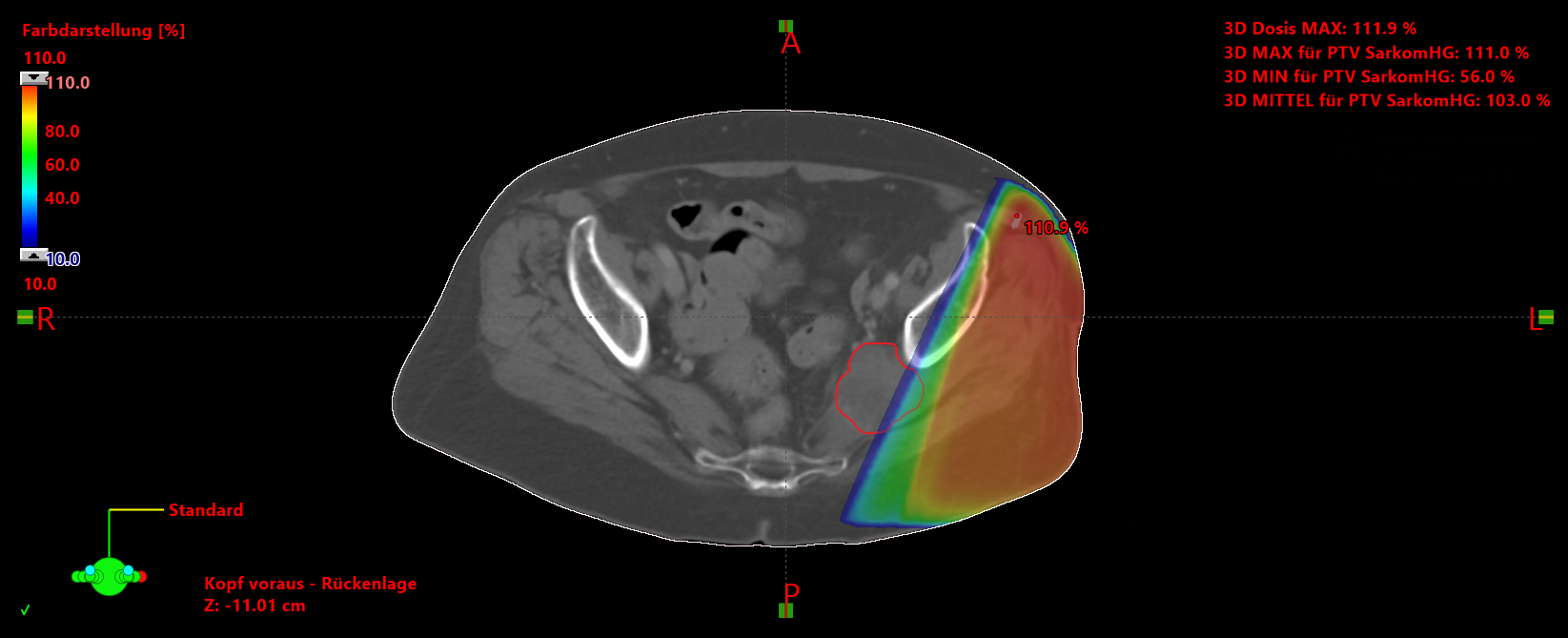


###### **Tables**

###### SM Table 1: Parameters for local recurrence after adj. RT with the distance of the recurrence to the original sarcoma and the isodose of the original irradiation field are given. R = resection status, IG-/IMRT = Image-guided/intensity-modulated radiation therapy, IGRT = Image-guided radiation therapy, IMRT = intensity-modulated radiation therapy, CTV long. = Clinical target volume longitudinal.

| **Age** | **Location** | **Histology** | **≥8cm** | **R** | **Grade** | **Dose (Gy)** | **Technique** | **PTV Long.** | | **Distance** | **Isodose** |
| --- | --- | --- | --- | --- | --- | --- | --- | --- | --- | --- | --- |
| 28 | thigh | Synovial sarcoma | yes | 1 | 3 | 66 | IG-/IMRT | 3.5 cm | 0 cm | | 90% |
| 22 | lower leg | other | yes | 1 | 3 | 44.80 | IGRT | 3.5 cm | 0 cm | | 90% |
| 83 | thigh | Pleomorphic sarcoma | yes | 1 | 2 | 60 | IG-/IMRT | unknown | 0 cm | | 95% |
| 56 | buttocks/hip | Synovial sarcoma | yes | 0 | 3 | 60 | IG-/IMRT | 3 cm | 3 cm | | 25% |

###### SM Table 2: surgery complications rates

| **Wound complications after surgery and VAC** | No. | % of n = 92 |
| --- | --- | --- |
| all wound complications | 27 | 29.3 % |
| Requiring secondary operations or invasive  procedures for wound care | 23 | 25.0 % |
| prolonged dressing changes | 7 | 7.6 % |
| infections within 120 days of surgery | 7 | 7.6 % |
| use of vacuum-assisted closure | 5 | 5.4 % |
